# Significantly reduced CCR5-tropic HIV-1 replication *in vitro *in cells from subjects previously immunized with Vaccinia Virus

**DOI:** 10.1186/1471-2172-11-23

**Published:** 2010-05-18

**Authors:** Raymond S Weinstein, Michael M Weinstein, Kenneth Alibek, Michael I Bukrinsky, Beda Brichacek

**Affiliations:** 1 Biodefense Program, Department of Public and International Affairs, George Mason University, 10900 University Blvd., MS 1H8, Manassas, VA 20110, USA; 2 Department of Human Genetics, University of California at Los Angeles, Gonda (Goldschmied) Neuroscience and Genetics Research Center, 695 Charles E. Young Drive South, Box 708822, Los Angeles, CA 90095, USA; 3 AFG Biosolutions, 9119 Gaither Rd., Gaithersberg, MD 20877, USA; 4 Department of Microbiology, Immunology and Tropical Medicine, The George Washington University, Ross Hall, Suite 734, 2300 Eye St., N. W., Washington, DC 20037, USA; 5 Department of Microbiology, Immunology and Tropical Medicine, The George Washington University, Ross Hall, Suite 734, 2300 Eye St., N. W., Washington, DC 20037, USA.; 6 Current address: National Cancer Institute, National Institutes of Health, Bethesda, MD, USA

## Abstract

**Background:**

At present, the relatively sudden appearance and explosive spread of HIV throughout Africa and around the world beginning in the 1950s has never been adequately explained. Theorizing that this phenomenon may be somehow related to the eradication of smallpox followed by the cessation of vaccinia immunization, we undertook a comparison of HIV-1 susceptibility in the peripheral blood mononuclear cells from subjects immunized with the vaccinia virus to those from vaccinia naive donors.

**Results:**

Vaccinia immunization in the preceding 3-6 months resulted in an up to 5-fold reduction in CCR5-tropic but not in CXCR4-tropic HIV-1 replication in the cells from vaccinated subjects. The addition of autologous serum to the cell cultures resulted in enhanced R5 HIV-1 replication in the cells from unvaccinated, but not vaccinated subjects. There were no significant differences in the concentrations of MIP-1α, MIP-1β and RANTES between the cell cultures derived from vaccinated and unvaccinated subjects when measured in culture medium on days 2 and 5 following R5 HIV-1 challenge.

**Discussion:**

Since primary HIV-1 infections are caused almost exclusively by the CCR5-tropic HIV-1 strains, our results suggest that prior immunization with vaccinia virus might provide an individual with some degree of protection to subsequent HIV infection and/or progression. The duration of such protection remains to be determined. A differential elaboration of MIP-1α, MIP-1β and RANTES between vaccinated and unvaccinated subjects, following infection, does not appear to be a mechanism in the noted protection.

## Background

A number of studies [1-4] have examined the origins of the human immunodeficiency virus (HIV) epidemic. Using epidemiological analyses and computer modelling, they have suggested that HIV-1 arose sometime around 1931 (1915-1941) from the simian immunodeficiency virus (SIV_cpz_) found in chimpanzees (Pan troglodytes troglodytes) of sub-Saharan, western central Africa, while HIV-2 is estimated to have independently arisen in western Africa about a decade later, 1940 ± 16 years, from the SIV (SIV_sm_) of sooty mangabeys (Cercocebus atys).

Beginning in the mid to late 1950s, both types of HIV entered a phase of exponential spread, first within Africa and then around the world. Wars, the reuse of unsterilized needles and other medical equipment in Africa during the 1950s and 1960s, and the contamination of early batches of polio vaccine in the 1950s have all been suggested as possible explanations for the emergence and spread of HIV. However, all of these theories have been either disproved or do not sufficiently explain the behaviour of the HIV pandemic [5-7]. The reasons behind HIV's sudden emergence and the mechanisms underlying its unique and highly successful adaptation to humans have yet to be elucidated. Even with the development of effective antiretroviral drugs, HIV continues to affect tens of millions of victims throughout the world and to ravage most of Sub-Saharan Africa as well as many large areas in Asia and Eastern Europe. The search for an effective HIV vaccine has thus far been intensive, expensive and fruitless.

The eradication of smallpox and the cessation of worldwide vaccinia-based vaccination programs--events that occurred in the mid-20^th ^century--have not been previously explored as a potential factor in the emergence and rapid spread of HIV. The suggestion that the progression of HIV-1 infection may be mitigated by an unrelated viral co-infection is not new. Co-infection with human herpesvirus 6 or 7 (HHV-6 or HHV-7) [8,9], GB virus C (GBV-C) [10], dengue fever virus [11], or the *paramyxovirus *responsible for measles [12,13] has been shown to mediate an inhibition of HIV-1 *in vivo *or *in vitro*. This inhibition appears to be mediated through the upregulation of CC chemokine receptor 5 (CCR5)-specific ligands and other cytokines, or by the downregulation of CD4 in the case of HHV-7. When the co-infecting virus can no longer be detected in the host, the protective effect seems to disappear in most cases.

One possible mechanism for the proposed relationship between HIV and pox viruses comes from the well known exploitation of CCR5 by HIV as a co-receptor to initiate infection in CD4+ lymphocytes and mononuclear cells [14,15]. Individuals homozygous for the CCR5Δ32 mutation--a null mutation of CCR5--are highly resistant to infection with HIV-1 [16,17]. Growing evidence suggests that many pox viruses, including vaccinia and variola require the presence of CCR5 as a permissive factor to generate a successful infection of some cells and preferentially infect CCR5-positive T cells [18-21]. As a consequence, it is possible that infection with some poxviruses may alter the expression of CCR5 on cell surfaces and/or the production of CCR5-specific ligands. Such events might interfere with a concurrent or subsequent infection by HIV-1.

Based on these data, we hypothesized that vaccinia immunization might confer some protection against initial HIV infection and possibly even disease progression. To test this hypothesis, we compared, *in vitro*, the susceptibility of peripheral blood mononuclear cells (PBMCs) from 10 vaccinia naïve subjects to those of 10 subjects immunized against smallpox no less than 3 and no more than 6 months prior to this study.

## Methods

### Subject selection

This study was approved by the institutional review boards of George Mason University, George Washington University and Potomac Hospital, and was conducted in accordance with the Helsinki Declaration. All subjects received both verbal and written informed consent and were told that results would be used in a study for potential publication. Twenty healthy volunteers were chosen from a group of naval personnel with a range in age of 19 to 41 years. Subjects included male and female, and white and non-white individuals. All subjects had a similar mix of previous immunizations with the exception that 10 subjects had been immunized with Dryvax (Wyeth) within the previous 3 to 6 months, and 10 subjects were vaccinia naive. Successful vaccination was confirmed by repeated visual inspections demonstrating the expected progression of the vaccination site. All subjects had a negative HIV test within the previous year. Two tubes of heparinized blood and one serum separator tube were collected. All blood samples from all subjects were drawn within 6 hours of each other and were immediately processed to separate the PBMCs using standard methods of Ficoll-Hypaque centrifugation [22,23]. After the cell cultures were started one vaccinated subject was dropped from the study because we learned that this individual had been having recurrent outbreaks of localized cutaneous vaccinia for several months since the vaccination. Since this might indicate an underlying occult health problem or immune deficiency it made the subject unsuitable for our study.

### Cell culture preparation

PBMCs were centrifuged at 1200 rpm for 11 minutes and resuspended in RPMI tissue culture medium + 10% fetal calf serum + 10 μg/ml gentamicin at a concentration of 1-3 × 10^6 ^cells/ml with a final concentration of 2 × 10^6 ^cells/culture. Cell cultures were incubated at 37°C in a CO_2 _incubator for 2 days then either an R5 strain (HIV-1_ADA_) or an X4 strain (HIV-1_NL4-3_) was mixed with an equal volume of either culture medium or serum from each individual subject and then incubated on ice for 7 hours after which 175 μl of each mixture was inoculated into the appropriate autologous cell cultures. No specific culture activating substances were added. After an overnight incubation, the cell cultures were washed with the described culture medium to remove non-attached virions. 150 μl of supernatant were aspirated for RT and/or chemokine analysis from each culture tube on days 2, 5, 8, 10, and 13. Beginning on day 8, half of the medium was changed after each supernatant collection.

### Reverse Transcriptase (RT) analysis

The measurements of viral replication were performed by standard RT assays using tritium-labelled thymidine as described elsewhere [22].

### Chemokine analysis

Levels of MIP-1α, MIP-1β and RANTES in culture supernatants were determined by specific ELISA (R&D Systems, Minneapolis, MN) according to the manufacturer's protocol.

### Statistical Analysis

Student's two-tailed, paired *t *test was used to determine statistical significance.

## Results and Discussion

All results are based on RT analysis using tritiated thymidine, and are given in counts per minute (cpm/μL). Cultures with no HIV added served as the negative control for the determination of background radioactivity. All of these control cultures had mean RT values of less than 100 cpm/μL on all days with no difference between vaccinated and unvaccinated subjects.

**Figure 1:**
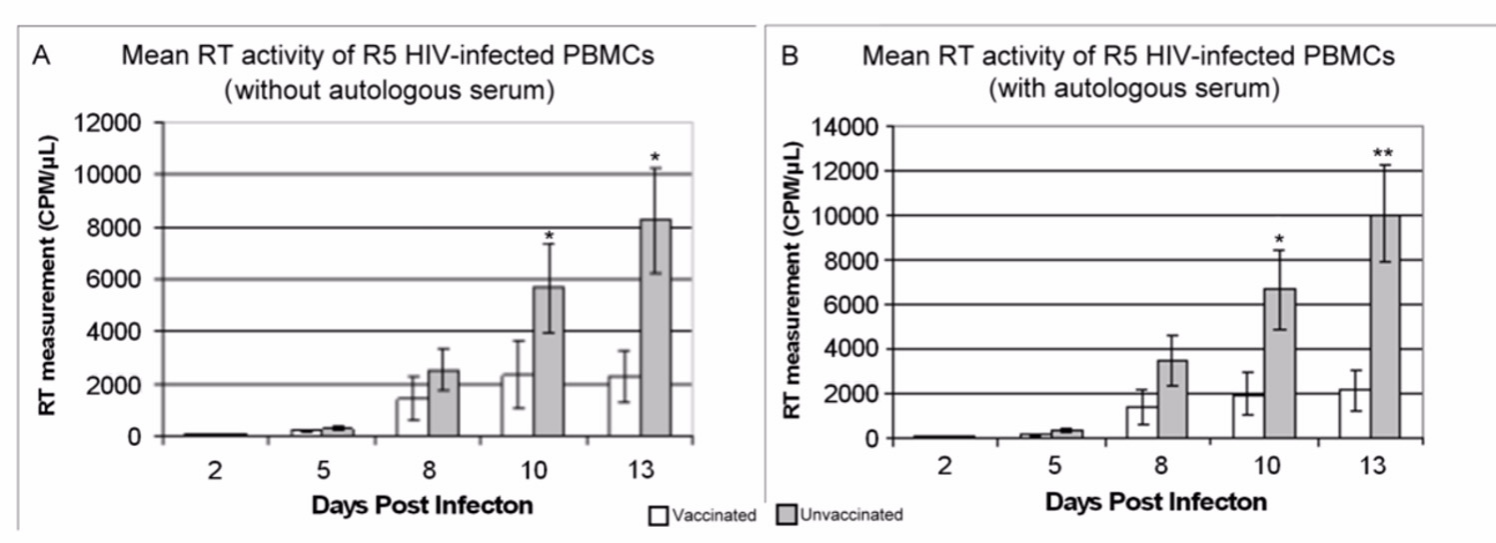
**Comparison of R5 HIV-1_ADA _replication in PBMC cultures from vaccinia naïve (N-10) and vaccinia vaccinated (N = 9) subjects**. Figure 1a shows cultures without and figure 1b shows cultures with pretreatment with autologous serum. A reduction in HIV replication can be seen on days 10 and 13. *p < 0.05 **p < 0.01.

Figure 1 shows the results from cultures infected with R5 HIV-1_ADA_. Without autologous serum pre-treatment (Figure 1a), a statistically significant mean reduction of HIV-1_ADA _replication was observed in cultures from vaccinated subjects when compared to unvaccinated subjects on days 10 (nearly 3 fold, p = 0.035) and 13 (4 fold, p = 0.017). Similar results were observed in cultures started with autologous serum pretreatment (Figure 1b), with a greater than 3 fold decrease by day 10 (p = 0.013) and a 5 fold decrease by day 13 (p = 0.008). R5 HIV-1 replication in cells from unvaccinated subjects with autologous serum pretreatment was greater on nearly all days compared to viral replication in cells from the same subjects without autologous serum. This is likely due to the activating effects of the serum on the cultured PBMCs. No such activation of viral replication occurred in cultures from vaccinated subjects with autologous serum pre-treatment, which remained nearly identical to that of cultures without the autologous serum, suggesting that vaccination prevented the enhancement of viral replication by the serum. This enhancement of HIV-1 replication in cultures infected in the presence of autologous serum in the unvaccinated subjects is responsible for the greater divergence seen between vaccinated and unvaccinated subjects in Figure 1b compared to Figure 1a.

**Figure 2:**
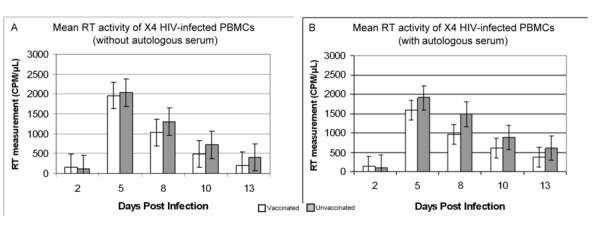
**Comparison of X4 HIV-1_NL4-3 _in PBMC cultures from vaccinia naïve (N = 10) and vaccinia vaccinated (N = 9) subjects**. Figure 2a shows cultures without and figure 2b shows cultures with pretreatment with autologous serum. No statistically significant difference between the vaccinated and unvaccinated subjects is found, however there is a trend toward reduced viral replication in the vaccinated subjects.

In cultures infected with X4 HIV-1_NL4-3 _(Figure 2) no statistically significant difference in viral replication between cells from vaccinated and unvaccinated subjects is observed although there is a trend toward reduction in HIV replication in vaccinated subjects. Pretreatment with autologous serum (Figure 2b) does not appear to make any difference in the replication of HIV-1_NL4-3 _when compared to non-pretreated cultures (Figure 2a). These findings suggest little if any effect by vaccinia immunization on replication of CXCR4-tropic HIV-1.

Within the narrow 3-6 month time frame of this study, there did not appear to be any relationship between the time since vaccination and the level of viral replication (data not shown). Cells from subjects vaccinated 6 months prior to the study showed similar reductions of viral replication to those in cells from subjects vaccinated 3 months before the study. This prolonged effect of vaccination is significantly different from that seen with other viruses known to inhibit HIV replication (measles, dengue fever virus and GBV-C), where such inhibition can only be demonstrated during the life of the actual co-infection and disappears when the co-infecting virus is no longer detectable [11,13,24]. Additionally, subsequent to this study, two of our co-authors independently repeated this study as part of a much larger investigation looking primarily at long term chemokine production after multiple immunizations [25]. In their study, they were able to demonstrate reduced CCR5-tropic HIV-1 replication in PBMC cultures from vaccinia immunized subjects vaccinated up to 14 months prior to their study. A statistically significant reduction in replication did not occur in cultures infected with a CXCR4-tropic HIV-1, although there was a trend toward reduced replication. Their results are nearly identical to those reported in this study, suggesting that an as-yet-to-be-identified suppressive effect on HIV replication is associated with vaccinia immunization, and persists for an extended time following vaccination, long after the vaccinia would be expected to be cleared from the host.

**Figure 3:**
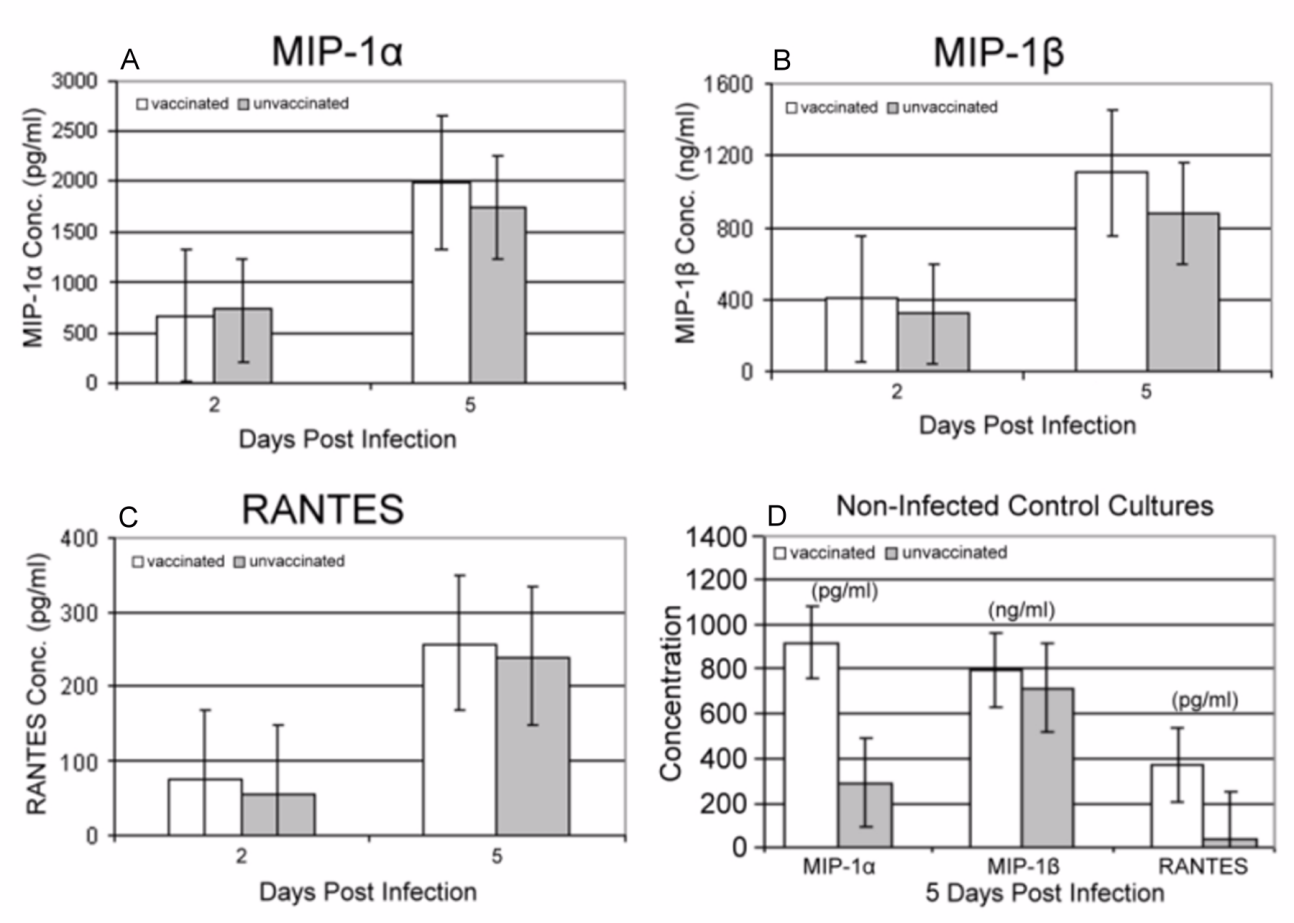
**Chemokine analysis in culture supernatants**. Comparison of MIP-1α (a), MIP-1β (b) and RANTES (c) release between the PBMCs from vaccinated (N = 9) and unvaccinated (N = 10) subjects on days 2 and 5 post culture inoculation with HIV-1_ADA_. No significant difference between the vaccinated and unvaccinated subjects is apparent. Figure 3d shows chemokine levels from non-infected control cultures (N = 2 for each group) on culture day 5. There is a trend toward a higher baseline chemokine production in the vaccinated subjects, though it is not statistically significant.

In addition, Brichacek, *et al. *in the above referenced study [25] demonstrated long term elevations of MIP-1α, MIP-1β and IL-8 in the serum of vaccinia immunized subjects compared to vaccinia naïve subjects. It is possible that these long term chemokine elevations may play some role in the observed resistance of PBMCs from vaccinated subjects to HIV-1_ADA _replication. In the present study we collected culture supernatant on days 2 and 5 following *in vitro *infection with HIV-1_ADA _for analysis of MIP-1α, MIP-1β and RANTES. Despite an observed trend towards higher levels of MIP-1α and MIP-1β in the cultures of vaccinated subjects, no statistically significant differences in the concentrations of those chemokines were found between the PBMC culture supernatants of vaccinated and unvaccinated subjects under our experimental conditions (Figure 3a, b, c). While it is possible that a long term elevation in baseline chemokine production may confer some protection against HIV infection and/or replication *in vivo*, an alteration in the ability of the PBMCs from vaccinated subjects to secrete an excess of these chemokines as a rapid response to an HIV-1 challenge does not appear to play a role under these culture conditions. Interestingly, the levels of these chemokines measured in the culture supernatant were generally much lower in the uninfected control cultures (Figure 3d), with the exception of RANTES which demonstrated levels equivalent to the HIV-1_ADA _infected cultures on culture day 5, but only for the vaccinated subjects. Though not statistically significant, there was also a trend toward higher baseline levels of all 3 chemokines in the vaccinated subjects, however the lack of statistical power may be related to the fact that only 2 vaccinated and 2 unvaccinated subjects underwent chemokine analysis for this control. The lack of statistical power prevents drawing any conclusions concerning this finding.

## Conclusions

Our findings support a heretofore unsuspected, yet significant beneficial interaction between HIV-1 and the pox virus vaccinia (and probably variola as well). Since the difference was only seen with CCR5-tropic HIV-1 and not with CXCR4-tropic HIV-1, the apparent resistance to HIV-1 in the vaccinated subjects is likely mediated, at least in part, by alterations in CCR5 or its ligands. However, our data suggest that this resistance is not mediated by a sudden post-infection surplus release of the chemokines MIP-1α, MIP-1β or RANTES in vaccinia immunized subjects when compared to unvaccinated subjects.

Most importantly, since primary HIV-1 infections are caused almost exclusively by CCR5-tropic HIV-1 strains [26] these results suggest that prior immunization with vaccinia virus might play a role in providing an individual with some degree of protection to subsequent HIV infection and/or disease progression. These results also provide some support to the hypothesis of a possible relationship between smallpox eradication and the still unexplained, sudden emergence of HIV-1. Further studies along these lines, involving larger groups of subjects are needed to substantiate our results and to fully elucidate the mechanism at work.

## Authors' contributions

RSW organized and designed the study, oversaw cell separation and culture preparation, performed the statistical analysis, and drafted the manuscript; MMW participated in planning the design and execution of the study, and in the writing of the manuscript; KA participated in overseeing cell separation and culture preparation, in designing and organizing the study, and in the writing of the manuscript; BB performed the *in vitro *infections and specimen collections, RT analysis, statistical analysis, participated in planning the execution of the study and participated in the writing of the manuscript; MIB participated the planning the execution of the study, *in vitro *infections, RT analysis, and participated in the writing of the manuscript. All authors read and approved the final manuscript.
